# A Core Outcome Set for Family-Centered Care in Neonatal Intensive Care Settings: An International eDelphi Study and Online Consensus Meeting

**DOI:** 10.3390/children13070862

**Published:** 2026-06-29

**Authors:** Cansel Kocakabak, Agnes van den Hoogen, Aurelia Abenstein, Anna Axelin, Karen M. Benzies, Livia N. Bonnard, Beatrix Callard, Marsha Campbell-Yeo, Linda S. Franck, Mary Anne Ryan, Pernilla Rönnholm, Patricia Schofield, Nicole R. van Veenendaal, Eleni Vavouraki, Anna Zanin, Jos M. Latour

**Affiliations:** 1 School of Nursing and Midwifery, Faculty of Health, University of Plymouth, Plymouth PL4 8AA, UK; patricia.schofield@plymouth.ac.uk; 2 Department Women and Baby, Neonatology, Wilhelmina Children’s Hospital, University Medical Centre Utrecht, Utrecht University, 3584 EA Utrecht, The Netherlands; ahoogen@umcutrecht.nl; 3 Global Foundation for the Care of Newborn Infants, 81379 Munich, Germany; aurelia.abenstein@gfcni.org; 4 Department of Nursing Science, University of Turku, 20520 Turku, Finland; anmaax@utu.fi; 5 Faculty of Nursing, University of Calgary, Calgary, AB T2N 416, Canada; benzies@ucalgary.ca; 6 Melletted a Helyem Egyesület Right(s) Beside You Association, 1013 Budapest, Hungary; livia.nagy@mellettedahelyem.hu; 7 Windhoek Central Hospital, Namibia International University of Management, Windhoek 10000, Namibia; cllbea001@myuct.ac.za; 8 School of Nursing, Faculty of Health, Dalhousie University, Halifax, NS B3H 4R2, Canada; marsha.campbell-yeo@dal.ca; 9 Institute for Health Policy Studies, University of California San Francisco, San Francisco, CA 94158, USA; linda.franck@ucsf.edu; 10 Infant Research Centre, University College Cork, T12 DC4A Cork, Ireland; maryanne.ryan@ucc.ie; 11 Prematurföreningen Mirakel, 416 74 Gothenburg, Sweden; pernilla.ronnholm@prematurmirakel.se; 12 Emma Children’s Hospital Department of Pediatrics, Amsterdam University Medical Center, University of Amsterdam, 1105 AZ Amsterdam, The Netherlands; n.r.vanveenendaal@amsterdamumc.nl; 13 Global Foundation for the Care of Newborn Infants, Associations for Premature Newborns, 11521 Athens, Greece; lela.vavouraki@gmail.com; 14 Department of Women, Children and Adolescents, University Hospital of Geneva, 1205 Geneva, Switzerland; anna.zanin@hug.ch; 15 Department of Nursing, Zhongshan Hospital, Fudan University, Shanghai 200031, China; 16 Curtin School of Nursing, Curtin University, Perth 6102, Australia

**Keywords:** family-centered care, core outcome set, neonatal intensive care units, infants, parents, health personnel, research, Delphi study, consensus meeting

## Abstract

**Highlights:**

**What are the main findings?**

- The core outcome set was established using a rigorous methodology, including comprehensive outcome identification, a three-round international eDelphi survey, and an expert online consensus meeting.
- A core set of 10 outcomes for family-centered care research in neonatal intensive care settings, including six outcomes for parents and four outcomes for infants, has been agreed upon by an international panel of healthcare professionals, parents and ex-neonatal patients.

**What are the implications of the main findings?**

- Implementation of this core outcome set may standardize outcome reporting, facilitating high-quality evidence synthesis and meta-analysis in family-centered care research in neonatal intensive care settings.
- Adoption of this core outcome set supports evidence-based family-centered care practices, ultimately improving parental and neonatal outcomes.

**Abstract:**

Background/Objectives: Family-centered care (FCC) in neonatal intensive care units (NICUs) can improve infant and family outcomes. Inconsistencies in outcome reporting across FCC trials limits the comparability of findings. Aim: To develop a core outcome set (COS) for evaluating FCC interventions in neonatal intensive care settings. Methods: A list of outcomes was generated through systematic reviews and stakeholder focus groups. A three-round eDelphi study with stakeholders was conducted, followed by an expert consensus meeting. Results: The reviews and focus groups identified 72 outcomes for round 1. Sixty-three healthcare professionals (HCP), 37 parents and five ex-NICU patients completed round 1, with 12 new outcomes suggested. In round 2, 54 HCP, 28 parents and four ex-NICU patients scored 84 outcomes, resulting in the exclusion of 12 low-importance outcomes. In round 3, 45 HCP, 28 parents, and two ex-NICU patients scored the remaining 72 outcomes. Overall, 71% of participants completed all three rounds. Round 3 yielded 48 outcomes that met the predefined consensus criteria and were taken forward to the expert consensus meeting. The final 10 COS outcomes included six outcomes related to parents, namely, bonding with their infant, participation in care, parental readiness for discharge, stress, shared decision-making, and parental knowledge of the infant’s care and treatment, and four outcomes related to infants, namely, infant pain and stress, growth, nosocomial infection, and length of NICU stays. Conclusions: A COS for FCC research and practice in neonatal intensive care settings has been established. Implementation of this COS may improve reporting consistency and strengthen evidence synthesis across FCC trails, thereby better informing care delivery in clinical practice.

## 1. Introduction

Preterm birth remains the leading cause of global neonatal mortality and morbidity, with surviving infants often requiring neonatal intensive or special care worldwide [1]. Although advances in neonatal care have substantially improved survival, many infants may experience developmental and physiological challenges later in life [2]. An infant’s admission to a neonatal intensive care unit (NICU) is distressing for parents [3]. A NICU admission may have long-term effects on both infants and their parents, underscoring the need for interventions that support infant health and parental well-being. Infants may experience adverse neurodevelopmental, behavioral, and social outcomes, whereas parents are at increased risk of psychological distress, including stress and anxiety [4].

Family-centered care (FCC) is a philosophy of care that emphasizes the importance of partnership between parents and healthcare professionals in the care and management of infants [5]. A range of FCC interventions has been implemented in NICUs, resulting in improvements in parental well-being, parent–infant bonding, and infant health and developmental outcomes [6,7]. However, FCC interventions and outcomes reported in studies are highly diverse and inconsistently defined [8,9]. This heterogeneity limits standardization and poses a significant challenge to the synthesis of findings in meta-analysis of FCC studies [10]. Consequently, the evidence base for FCC is weakened, which hinders the translation of research findings into routine neonatal practice. The absence of standardized outcome reporting makes it difficult to identify the most effective FCC interventions and prioritize their implementation across neonatal intensive care settings. This can lead to challenges in comparing interventions, developing strong evidence-based recommendations, and allocating health resources effectively. Given the increasing global awareness of FCC, there is an urgent need for standardized outcome reporting. The development of a core outcome set (COS) may help to address these challenges.

A COS is an agreed and standardized minimum set of outcomes, developed through consensus among key stakeholders, that should be measured and reported in clinical trials evaluating a specific intervention [11]. The use of a COS may reduce heterogeneity in outcome reporting, improve comparability across studies, and facilitate evidence synthesis and meta-analysis. To date, no COS has been developed for FCC interventions in neonatal settings. Therefore, the aim of the COUSIN (core outcome set and outcome measures of family-centered care in neonatal care) study was to develop an internationally agreed core outcome set for evaluating family-centered care interventions and practices in neonatal intensive care settings.

## 2. Materials and Methods

This study was conducted in an international context, including participants with neonatal intensive care experience and expertise from multiple backgrounds, countries, and settings. The study was prospectively registered with the COMET initiative (Registration number 2002; https://www.cometinitiative.org/Studies/Details/2002, accessed on 27 June 2026). Ethical approval was obtained from the University of Plymouth Research Ethics Committee (reference number: 6146). The COS was developed following the methodology described in the COMET Handbook [12] and the Core Outcome Set-Standards for Development (COS-STAD) [13]. Reporting of this COS followed the Core Outcome Set—Standards for Reporting (COS-STAR) guideline [14].

### 2.1. COUSIN Study Steering Group

The COUSIN Steering Group was established in October 2021 and included a parent representative and patient advocate from the Global Foundation for the Care of Newborn Infants (GFCNI) (A.A.), an assistant professor and expert in neonatal care and research (A.v.d.H.), a doctoral researcher (C.K.), professors and experts in neonatal intensive care (A.A., M.C.Y., L.S.F., and J.M.L.), and a neonatologist (N.v.V.).

### 2.2. COUSIN Study Design

**Figure 1:**
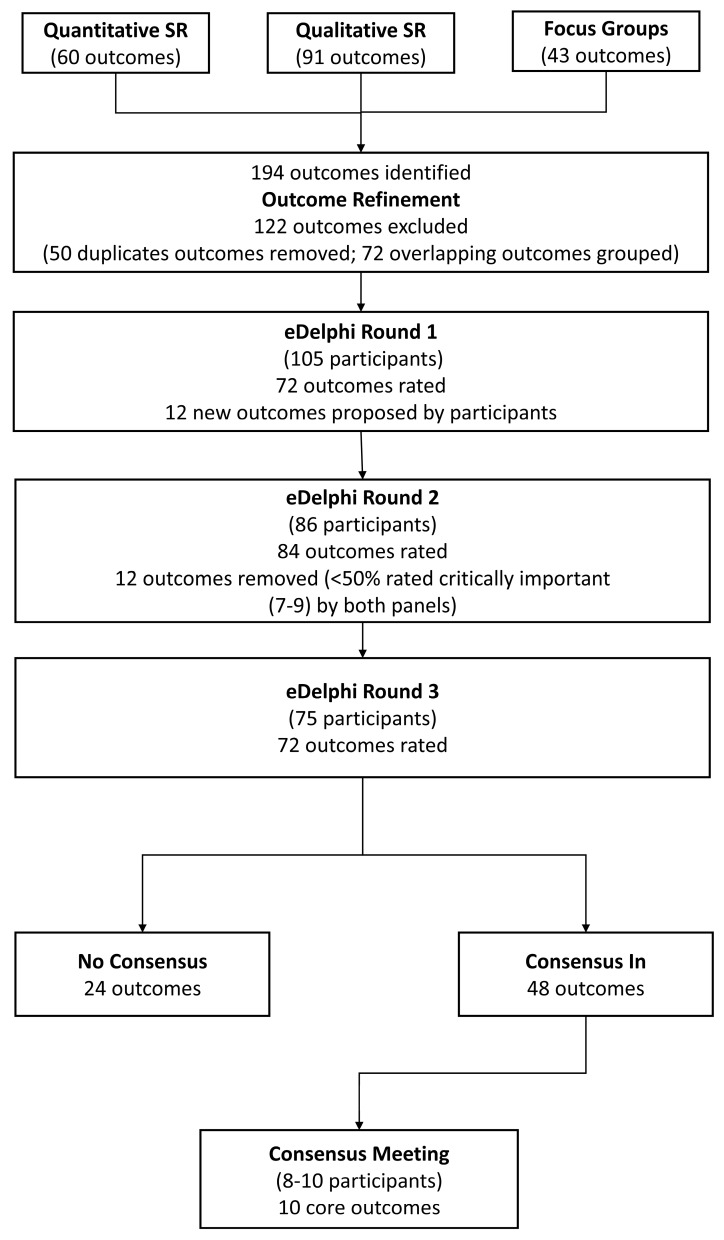
Flow of outcome identification, eDelphi rounds, and consensus process for the development of the core outcome set. Figure legend: SR, systematic review.

The COS was developed in three phases: (1) identification of outcomes, (2) a three-round eDelphi survey, and (3) an online consensus meeting (Figure 1).

### 2.3. Phase 1: Identification of Outcomes

Phase 1 aimed to generate a comprehensive list of potential outcomes for FCC interventions in neonatal intensive care settings. Outcomes were identified using three data sources: (1) a quantitative systematic review was conducted to identify outcomes reported in studies evaluating FCC interventions [15]; (2) a qualitative systematic review was conducted to synthesize parents’ experiences related to FCC and to identify outcomes derived from these experiences [16]; and (3) focus group discussions were conducted to explore the views of former neonatal patients, parents, and healthcare professionals with experience in FCC and to elucidate outcomes that were important to each constituency [17].

#### Outcome List Refinement

The research team, comprising two supervisors (J.M.L., A.v.d.H.), a parent representative (A.A.) from GFCNI, and a PhD student (C.K.), held two meetings to refine the outcome list for the eDelphi survey. To ensure clarity for participants, these outcomes were categorized into parent, infant, and staff domains.

### 2.4. Phase 2: eDelphi Study

Participants completed a three-round eDelphi survey to rate the importance of the outcomes. The surveys were distributed online using the Joint Information Systems Committee (JISC, Bristol, UK) (https://app.onlinesurveys.jisc.ac.uk/dashboard/plymouth, accessed on 27 June 20260) v. 3 survey tool, which is compliant with the UK General Data Protection Regulation (GDPR). The surveys were reviewed and tested by the research team before each round to ensure clarity and functionality.

In each round, participants rated the importance of outcomes using a 9-point Likert scale, in accordance with the Grading of Recommendations Assessment, Development, and Evaluation (GRADE) guideline [18]. An “unable to answer” option was provided for participants who considered themselves unable to assess the outcome. Each numerical score was accompanied by a descriptive label in order to support the correct interpretation of the scale (Electronic Supplemental Material, Figure S1).

#### 2.4.1. Participant Recruitment

Participants were recruited through parent and professional organizations:

- Global Foundation for the Care of Newborn Infants (GFCNI);
- Council of International Neonatal Nurses (COINN);
- European Society of Pediatric and Neonatal Intensive Care (ESPNIC);
- European Society of Pediatric Research (ESPR).

These organizations disseminated invitations to the first round of the eDelphi survey through newsletters and their email networks.

#### 2.4.2. Stakeholder Panels

In line with COS-STAD recommendations [13], two stakeholder groups were included: (1) parents and former neonatal patients with FCC experience, and (2) healthcare professionals with FCC experience in neonatal practice and research. As no standardized sample size is recommended for Delphi studies [19], a minimum target sample size of 40 participants for each stakeholder group was set.

#### 2.4.3. Feedback Between Rounds

In round 1, participants were provided with an optional open-text box to suggest additional outcomes not identified in Phase 1. All suggested outcomes were reviewed by the research team and eligible outcomes were included in round 2. Before rounds 2 and 3, participants received anonymized feedback for each outcome, presented as the distribution of scores across important categories (1–3, 4–6, and 7–9), together with the mean score for each stakeholder group (Electronic Supplemental Material, Figure S2). No outcomes were removed between rounds 1 and 2, allowing participants to review group feedback before re-scoring. Between rounds 2 and 3, outcomes were removed in accordance with pre-specified consensus criteria to reduce participant burden and minimize attrition.

#### 2.4.4. Consensus Definition

Consensus was defined using the 70/15% rule: outcomes were considered ‘consensus in’ if ≥70% of participants rated the outcome within the critical importance range (scores 7–9), and ≤15% rated it as limited importance (scores 1–3). Outcomes were considered ‘consensus out’ if ≥70% rated the outcome as limited importance (score 1–3) and ≤15% rated it within the critical importance range (scores 7–9). All other outcomes were classified as ‘no consensus’ [11]. To minimize the risk of excluding outcomes that were important to one stakeholder group, any outcome rated within the critical importance range (scores 7–9) by at least 50% of participants in at least one stakeholder panel was retained between rounds 2 and 3, even if it did not meet the overall consensus criteria.

### 2.5. Phase 3: Consensus Meeting

One online international expert consensus meeting was organized for 15 October 2025 to finalize the COS for FCC. This was conducted online, offering a practical approach while also widening geographical reach to encompass international participants. The meeting was chaired by the PhD student (CK) and included the supervisor (JML) and a note taker, and was held via Zoom (San Jose, CA, USA; https://zoom.us/, accessed on 15 October 2025), lasting three hours. Participants who had completed round 3 and expressed an interest were invited to participate, alongside members of the COUSIN Study Steering Group. All outcomes from round 3 of the eDelphi reaching ≥70% agreement criteria progressed to the final consensus meeting for discussion and final votes. This list of outcomes was shared with participants prior to the meeting (Electronic Supplemental Material, Tables S1–S3). During the meeting, outcomes were presented from highest to lowest percentage of agreement. Participants were encouraged to ask questions and discuss their views. This was followed by two rounds of anonymous voting using Mentimeter^®^ (AB, Stockholm, Sweden; https://www.mentimeter.com/, accessed on 15 October 2025), indicating ‘Yes’ (this outcome should be included in the COS) or ‘No’ (this outcome should not be included) on each outcome. To ensure that only outcomes with a high level of agreement were included in the final COS, a higher threshold (≥80%) was applied in each round of the consensus meeting voting. This threshold was used exclusively for final ratification of outcomes into the final COS, and is different from the consensus criteria applied during the eDelphi rounds.

## 3. Results

A total of 194 possible outcomes were identified, including 60 outcomes from the quantitative systematic review, 91 outcomes from the qualitative systematic review, and 43 outcomes from focus group discussions. Following outcome refinement, overlapping outcomes were merged, resulting in a final list of 72 outcomes for eDelphi (Figure 1).

### 3.1. Participant Characteristics

A total of 105 participants from 39 countries completed round 1 of the eDelphi survey and were invited to participate in subsequent rounds. No new participants were added in rounds 2 and 3. The eDelphi study started with 37 parents, five former neonatal patients, and 63 healthcare professionals. The largest number of healthcare professionals were nurses (n = 39; 62%), followed by doctors (n = 16; 25%), and allied health professionals (n = 8; 13%).

Overall attrition from round 1 to round 3 was 29%, with 75 of 105 participants completing all three rounds. Attrition rates were similar across stakeholder groups, with 28.6% attrition among parents/former neonatal patients and healthcare professionals. Participant characteristics across eDelphi rounds by stakeholder group are summarized in Table 1.

### 3.2. eDelphi Surveys

Round 1 was open from 7 May to 30 June 2025. The survey included 72 outcomes, including 36 parent outcomes, 28 infant outcomes, and eight healthcare professionals outcomes. Participants added 65 comments regarding potential additional outcomes. From these suggestions, 12 new outcomes (three parent outcomes, six infant outcomes, and three staff outcomes) were added for round 2, and two outcomes were revised as to their wording.

Round 2 was open from 11 July to 21 August 2025. In this round, 86 (82%) participants responded: healthcare professionals (n = 54); and parents (n = 28) and former neonatal patients (n = 4). The respondents scored 84 outcomes (39 parent outcomes, 34 infant outcomes, and 11 staff outcomes). Both stakeholder groups excluded 12 outcomes after round 2 because 50% or fewer participants rated them within the critical importance range (scores 7–9). Round 3 was open from 26 August to 1 October 2025. In this round, 75 participants (healthcare professionals (n = 45); and parents (n = 28) and former neonatal patients (n = 2)) responded. The respondents scored 72 outcomes (36 parent outcomes, 25 infant outcomes, and 11 staff outcomes). Following round 3, 48 outcomes reached consensus, and 24 outcomes did not reach consensus.

The healthcare professionals panel changed their scores after reviewing aggregated feedback, whereas no notable changes were observed in the parents and former neonatal patients panel (Electronic Supplemental Material, Table S4). For parental outcomes, 28 out of the 39 outcomes showed a change in scoring distributions, while the remaining 11 outcomes (sleep deprivation, anticipatory grief, self-blame, parental guilt, parental preparedness for NICU admission, parental comfort, confidence in breastfeeding, perceived support from family and friends, and perceived family functioning) did not change from ‘no consensus’. Three outcomes within the parent domain were excluded at the end of round 2 and were not carried forward to round 3 (Table 2).

Across domains, infant outcomes were generally rated lower compared to parent and staff outcomes. Only three of the infant outcomes (growth and development, neurodevelopment, and length of NICU stay) achieved consensus by all three rounds. Nine infant outcomes (formula feeding, combination feeding, time to full gastrointestinal feeding, duration of nasogastric tube retention, duration of total enteral nutrition, hospital expenses, duration of antibiotic use, infiltration of peripheral intravenous, and exclusive breastfeeding at discharge) were excluded at the end of round 2 and were not carried forward to round 3.

Outcomes within the healthcare professional domain achieved consensus early in the eDelphi process and maintained high proportions of critical ratings (7–9) across subsequent rounds. By the end of round 3, all 11 outcomes in the healthcare professional domain (staff attitudes, staff confidence, staff relationship with parents, staff workload, staff competency, staff responsiveness parental needs, staff satisfaction, delivery of continuity care, staff health and well-being, staff training and knowledge of FCC, staff turnover, intention to leave) had reached consensus.

### 3.3. Consensus Meeting

Twenty-four participants expressed interest in attending the consensus meeting. Email invitations with the Zoom link were distributed. Of the 24 participants, seven accepted the invitation, three declined, 13 did not respond, and one selected tentative availability. Eleven participants attended the online consensus meeting, with 8–10 participants participating in each voting round. The consensus meeting chair and note-taker did not participate in voting. Three steering group members participated in the voting. The number of votes varied across outcomes because not all attendees voted on every outcome (Electronic Supplementary Material, Table S5). The healthcare professionals included four nurses and three doctors (Table 3).

During the online consensus meeting, the first round of voting was conducted on the 48 outcomes that had reached consensus in the eDelphi survey (Electronic Supplemental Material, Table S5). In the first-round voting of the online consensus meeting, 13 out of 48 outcomes (bonding with infant, infant pain, staff training and knowledge of FCC, participation in care, infant stress, parental readiness for discharge, stress, parent–staff communication, shared decision-making, parental knowledge of infant’s care and treatment, growth and development, nosocomial infection, length of NICU stay) received ≥80% agreement for inclusion in the COS. Parental presence achieved 70% agreement in the first-round voting; however, following discussion, experts agreed that this outcome warranted further consideration and retained it for a second round of voting.

During the subsequent discussion, participants suggested combining the “infant pain” and “infant stress” outcomes into a single outcome, “infant pain and stress.” Although conceptually distinct, experts considered these outcomes to have substantial overlap in their assessment. The outcome “parental presence,” although it did not meet the ≥80% agreement threshold, was retained for the second round of voting following participants’ requests. In the second round of voting, four outcomes (staff training and knowledge of family-centered care, parental readiness for discharge, parent–staff communication, and parental presence) were excluded because they did not meet the predefined ≥80% agreement threshold. However, following further discussion, the “parental readiness for discharge” outcome was re-voted upon and subsequently included in the final COS.

During the final consensus meeting, provision of the mother’s own milk was suggested as a new outcome. This outcome was not identified during the outcome identification phases or during the eDelphi rounds, maybe because related outcomes such as “exclusive breastfeeding,” “breastfeeding,” and “mother’s confidence in breastfeeding” were already included. Provision of the mother’s own milk was considered as an important outcome by some of the consensus experts but was not included in the final COS. The final COS included 10 outcomes across infant and parent domains (Table 4).

## 4. Discussion

An internationally agreed COS was developed for evaluating FCC interventions in neonatal intensive care settings. A total of 194 possible outcomes were identified, and the list was refined to 72 outcomes, with a final set of 10 core outcomes that were ratified by international stakeholders. A critical gap in outcome standardization has been identified in previous systematic reviews of FCC [10,20]. Our final COS is recommended for use in future evaluations of FCC interventions trials in neonatal settings.

Several outcomes within the COS aligned well with existing international and national guidance. Parental participation in care was retained in the final COS and is explicitly recommended in international FCC guidelines and recommendations [21,22]. The inclusion of this outcome in the final COS underscores its importance as a fundamental component of FCC and highlights the central role of parents in FCC interventions. This is supported by evidence suggesting that parental participation in care is associated with improving infant neurodevelopment, reduced morbidity, and enhanced parental well-being [20,23]. Participation in care requires parental presence; however, physical presence is not the sole indicator of active involvement in caregiving [24]. While FCC principles advocate zero separation between parents and infants, parents are recognized as primary caregivers rather than visitors. Although parental participation in care may not always be feasible across international clinical practice due to differences in cultural contexts, organizational structures, environmental constraints, and staff attitudes [25], parental presence remains fundamental for shared decision-making. Shared decision-making reflects a collaborative partnership between parents and healthcare professionals that underpins FCC and is acknowledged as a main component of FCC [26]. The effectiveness of FCC has been associated with greater parent–infant bonding [27] and a shorter NICU stay [28]. Collectively, this evidence underscores the importance of these outcomes and supports their inclusion as core outcomes in the final COS.

The outcome “nosocomial infection” received comparatively lower ratings across the eDelphi rounds yet was retained in the final COS. Parental presence and participation in neonatal intensive care settings have traditionally been restricted due to concerns about infection risk [29]. However, emerging evidence suggests that FCC practices may contribute to infection prevention rather than increase infection risk [30]. Measuring the “nosocomial infection” outcome within the COS is critical for informing policy decisions, addressing persistent clinical concerns, and supporting safe implementation of FCC in neonatal practice.

Healthcare professionals-related outcomes were highly rated throughout the eDelphi process but were not retained in the expert voting for the final COS during the consensus meeting. While these outcomes are central to the implementation of FCC, they may be more appropriately conceptualized as implementation or process outcomes rather than core effectiveness outcomes, as they reflect the quality of FCC delivery. Nevertheless, their prominence in stakeholder scoring highlights the critical role of staff in the effective implementation of FCC. The existing literature emphasizes that successful FCC interventions require appropriate staff training, attitude change, and organizational support [31].

An existing neonatal core outcome set developed by Webbe et al. (2019) [32] focused primarily on survival and major morbidity outcomes. In contrast, our COS addresses outcomes specific to FCC, including relational and care-process outcomes, and no identical outcomes were identified between the two COSs. This difference reflects the distinct scopes of the two COSs, which targeted different aspects of neonatal care. Importantly, incorporating outcomes reflecting parental experiences alongside clinical indicators aims to ensure that FCC research remains meaningful, relevant, and applicable to practice. This contrast also reflects distinct priorities in neonatal research. A previous scoping review [33] in NICU identified a range of infant and parent outcomes, several of which aligned with those retained in our COS. However, the review mapped published research rather than presenting a consensus-based list of agreed outcomes for the field. Recently, a scoping review highlighted the limited evidence regarding long-term neonatal and parental outcomes [9]. Consistent use of the COS developed in the present study may facilitate the generation of more robust evidence on both short- and long-term impacts of FCC, as well as the sustainability of FCC practices in neonatal care.

### Limitations

This study has several strengths, including strong engagement across eDelphi rounds, with low attrition and the inclusion of diverse international stakeholders. Despite these strengths, several limitations remain. The eDelphi surveys were conducted in English, which may have limited participation from non–English-speaking stakeholders despite efforts to include diverse settings. Additionally, the conceptual heterogeneity of FCC interventions may have influenced participants’ interpretation of certain outcomes, potentially contributing to variability in scoring.

Although the number of participants at the consensus meeting was small, it was consistent with previously published COS studies in neonatology [32,34]. However, participants in the final consensus meeting were predominantly from high-income countries, with limited representation from low- and middle-income countries, despite their disproportionately higher neonatal burden. Consequently, the applicability of the COS should be interpreted cautiously when implemented in low- and middle-income countries. In addition, although former neonatal patients participated in the eDelphi process, they were not represented at the final consensus meeting, which may have limited the extent to which their perspectives were reflected in the final COS. Furthermore, only 8–10 participants voted in each round in the consensus meeting; individual votes had a relatively large influence on whether outcomes met the predefined criteria. However, outcomes were discussed collectively, and where participants considered further clarification necessary, additional discussion and re-voting were undertaken before final decisions were made.

## 5. Conclusions

This study developed an internationally agreed upon COS for evaluating FCC interventions and practices in neonatal intensive care settings. To address heterogeneity in outcome reporting, this COS provides a standardized and stakeholder-informed set of outcomes for future FCC research. Consistent implementation of this COS may enhance comparability across FCC trials and strengthen evidence synthesis. Furthermore, national and international neonatal intensive care settings databases may consider including the 10 core outcomes, which may facilitate the generation of robust evidence to inform clinical practice and support the development of strong evidence-based guidelines for neonatal FCC. Future work should focus on developing a core outcome measurement instrument and establishing standardized definitions for these outcomes included in the final COS. In addition, defining outcomes with standard terminology and standardized definitions requires careful consideration.

## Figures and Tables

**Table 1:** Participants characteristics across eDelphi rounds, by stakeholder group.

Characteristics	Round 1			Round 2			Round 3		
	P n = 37	FNP n = 5	HCP n = 63	P n = 28	FNP n = 4	HCP n = 54	P n = 28	FNP n = 2	HCP n = 45
**Gender**									
Female	34	5	54	25	4	45	25	2	39
Male	3	0	9	3	0	9	3	0	6
**Age group**									
18–30	1	1	3	0	1	3	0	1	3
31–40	11	3	14	9	2	14	9	0	13
41–50	18	1	17	14	1	15	14	1	8
51–60	7	0	20	5	0	16	5	0	16
>60	0	0	8	0	0	6	0	0	5
Missing	0	0	1	0	0	0	0	0	0
**Ethnicity**									
White	29	4	44	22	3	38	22	1	35
Asian	1	0	7	0	0	6	0	0	3
Black	2	0	6	1	0	4	1	0	2
Mixed/Multiple ethnic group	2	1	2	2	1	2	2	1	1
Hispanic	1	0	1	1	0	1	1	0	1
Missing	0	0	1	0	0	1	0	0	1
Other	2		2	2	0	2	2	0	2
**Region**									
Europe	22	1	35	18	1	29	18	1	27
North America	8	4	1	5	3	1	5	1	1
South America	0	0	1	0	0	1	0	0	1
Oceania	4	0	11	3	0	11	3	0	10
Africa	2	0	8	2	0	5	2	0	4
Asia	1	0	5	0	0	5	0	0	1
Missing	0	0	2	0	0	1	0	0	1

Abbreviations: P = parents; FNP = former neonatal patients; HCP = healthcare professionals; n = number of participants.

**Table 2:** Distributions of scores (1–3; 4–6; 7–9) across eDelphi rounds.

		Round 1				Round 2				Round 3			
		1–3 %	4–6 %	7–9 %	r1 Consensus	1–3 %	4–6 %	7–9 %	r2 Consensus	1–3 %	4–6 %	7–9 %	r3 Consensus
Domains	Outcomes												
Parent	Stress	0	25	75	IN	1	7	92	IN	1	11	88	IN
	Depression	0	29	71	IN	0	14	86	IN	0	23	77	IN
	Anxiety	0	25	75	IN	2	11	87	IN	1	17	81	IN
	Sleep deprivation	2	34	64	No Consensus	6	30	64	No Consensus	4	44	52	No Consensus
	Post-traumatic stress disorder	4	21	76	IN	1	24	74	IN	0	30	70	IN
	Anticipatory grief	5	35	60	No Consensus	5	39	56	No Consensus	3	47	50	No Consensus
	Self-blame	5	34	61	No Consensus	4	41	56	No Consensus	3	45	52	No Consensus
	Parental guilt	4	30	66	No Consensus	2	35	63	No Consensus	3	44	53	No Consensus
	Coping with emotions	1	24	76	IN	1	31	67	No Consensus	0	27	73	IN
	Coping through religious or spiritual practice	15	32	53	No Consensus	19	53	28	OUT				OUT
	Parental competence of caring	2	23	75	IN	0	12	88	IN	0	8	92	IN
	Parental autonomy	0	32	68	No Consensus	1	21	78	IN	0	19	81	IN
	Self-efficacy	3	34	64	No Consensus	1	27	72	IN	0	17	83	IN
	Parental confidence in caring	0	23	77	IN	0	15	85	IN	0	11	89	IN
	Perceived parental role (maternal or paternal)	3	24	73	IN	1	20	79	IN	0	27	73	IN
	Participation in care	0	13	87	IN	0	5	95	IN	0	7	93	IN
	Parent–infant interaction	0	5	95	IN	0	4	97	IN	0	5	95	IN
	Parental presence	3	5	93	IN	0	12	88	IN	0	9	91	IN
	Skin-to-skin contact	0	8	92	IN	1	8	91	IN	1	5	93	IN
	Physically holding the infant	0	18	83	IN	0	16	84	IN	3	9	88	IN
	Bonding with infant	0	6	94	IN	0	4	97	IN	0	3	97	IN
	Separation	5	22	73	IN	1	17	82	IN	1	19	80	IN
	Parental satisfaction with care	1	30	69	No Consensus	2	24	73	IN	0	29	71	IN
	Parent–staff communication	1	12	87	IN	0	8	92	IN	0	15	85	IN
	Shared decision-making	1	21	78	IN	0	14	86	IN	0	16	84	IN
	Parental perception of staff support	3	29	68	No Consensus	0	26	74	IN	0	25	75	IN
	Parental trust in staff	1	14	85	IN	0	11	90	IN	0	9	91	IN
	Parental knowledge of infants’ care and treatment	1	26	74	IN	0	16	84	IN	0	16	84	IN
	Parental preparation for NICU admission	5	46	49	No Consensus	4	50	47	No Consensus	6	51	44	No Consensus
	Parental readiness for discharge	0	17	83	IN	1	7	92	IN	0	11	89	IN
	Parental ability to advocate for their baby	1	21	78	IN	0	23	77	IN	0	13	87	IN
	Parental comfort	1	39	60	No Consensus	4	44	52	No Consensus	1	45	53	No Consensus
	Quality of life	4	25	71	IN	0	22	78	IN	1	33	65	No Consensus
	Confidence in breastfeeding	4	30	67	No Consensus	2	31	66	No Consensus	1	37	61	No Consensus
	Perceived support from family and friends	7	50	43	No Consensus	4	48	49	No Consensus	4	64	32	No Consensus
	Perceived family functioning	3	42	55	No Consensus	2	51	47	OUT				
	Use of milk bank					17	49	34	OUT				
	Peer support					5	47	49	No Consensus	1	44	55	No Consensus
	Parent understanding of infant development					0	27	73	IN	0	23	77	IN
Infant	Mortality	14	18	67	No Consensus	6	29	65	No Consensus	5	24	71	IN
	Necrotizing enterocolitis	14	31	55	No Consensus	8	31	61	No Consensus	5	45	50	No Consensus
	Weight gain	2	34	64	No Consensus	2	28	70	IN	1	41	57	No Consensus
	Breastfeeding	4	25	71	IN	4	31	65	No Consensus	3	40	57	No Consensus
	Formula feeding	15	51	34	No Consensus	13	65	22	OUT				
	Combination feeding methods (Breast, formula and bottle)	12	41	47	No Consensus	6	57	37	OUT				
	Gastric tube feeding	13	45	42	No Consensus	6	52	42	No Consensus	6	58	37	No Consensus
	Time to full gastrointestinal feeding	7	46	48	No Consensus	6	52	42	OUT				
	Duration of nasogastric tube retention	13	53	34	No Consensus	10	58	32	OUT				
	Duration of total enteral nutrition	15	44	41	No Consensus	10	54	36	OUT				
	Feeding intolerance	15	42	43	No Consensus	9	47	44	No Consensus	7	51	43	No Consensus
	Growth and development	3	13	84	IN	0	11	90	IN	3	24	73	IN
	Duration of oxygen therapy	12	24	65	No Consensus	5	25	71	IN	3	40	58	No Consensus
	Bronchopulmonary dysplasia	15	28	57	No Consensus	7	31	61	No Consensus	4	44	51	No Consensus
	Retinopathy of prematurity	20	29	51	No Consensus	6	34	60	No Consensus	4	44	51	No Consensus
	Resuscitation	17	25	58	No Consensus	8	29	63	No Consensus	7	29	64	No Consensus
	Neurodevelopment	2	10	86	IN	0	6	94	IN	0	7	93	IN
	Sepsis	6	27	68	No Consensus	2	24	74	IN	4	24	72	IN
	Intraventricular hemorrhage	13	27	60	No Consensus	2	26	71	IN	3	22	75	IN
	Nosocomial infection	7	30	63	No Consensus	2	35	63	No Consensus	3	25	73	IN
	Length of hospital stay	4	26	71	IN	1	21	78	IN	1	31	68	No Consensus
	Length of NICU stay	4	25	71	IN	1	22	77	IN	3	27	71	IN
	Readmission	2	39	59	No Consensus	2	27	71	IN	1	32	67	No Consensus
	Hospital expenses	25	38	38	No Consensus	18	48	34	OUT				
	Duration of antibiotic use	21	35	43	No Consensus	13	54	33	OUT				
	Infiltration of peripheral intravenous	20	43	37	No Consensus	17	54	29	OUT				
	Severity of illnesses	11	29	60	No Consensus	6	27	67	No Consensus	1	31	68	No Consensus
	Infant stress	11	40	50	No Consensus	1	16	83	IN	0	8	92	IN
	Infant pain					0	16	84	IN	0	5	95	IN
	Exclusive breastfeeding at discharge					9	49	42	OUT				
	Use of non-pharmacological measures (massage, music)					12	43	45	No Consensus	4	36	60	No Consensus
	Comfort of infant					1	15	84	IN	0	15	85	IN
	Infant sleep quality					1	29	69	No Consensus	1	17	81	IN
	Hypoxic ischemic encephalopathy					14	30	57	No Consensus	4	41	55	No Consensus
Staff	Staff attitudes	2	21	77	IN	0	11	90	IN	0	11	89	IN
	Staff confidence	1	20	79	IN	0	11	90	IN	0	12	88	IN
	Staff relationship with parents	1	14	85	IN	0	13	87	IN	0	9	91	IN
	Staff workload	1	18	81	IN	1	9	90	IN	0	11	89	IN
	Staff competency	0	15	85	IN	0	9	91	IN	0	3	97	IN
	Staff responsiveness to parental needs	0	22	78	IN	1	19	80	IN	0	13	87	IN
	Staff satisfaction	5	30	66	No Consensus	1	27	72	IN	1	25	73	IN
	Delivery of continuity care	2	20	78	IN	0	16	84	IN	0	25	75	IN
	Staff health and well-being					2	29	69	No Consensus	1	23	76	IN
	Staff training and knowledge of family-centered care					1	9	90	IN	0	7	93	IN
	Staff turnover, intention to leave					5	27	69	No Consensus	4	24	72	IN

**Table 3:** Online consensus meeting participant characteristics.

Characteristics	Parents (n = 4)	Healthcare Professionals (n = 7)
**Gender**		
Female	4	7
**Age group**		
31–40	1	1
41–50	2	1
51–60	1	3
>60	0	2
**Ethnicity**		
White	3	7
Sami heritage	1	0
**Country**		
Canada	0	2
Netherlands	0	1
Cyprus	1	0
France	1	0
Greece	0	1
Italy	0	1
Ireland	0	1
Namibia	0	1
Sweden	1	0
Germany	1	0

**Table 4:** Final core outcome set for family-centered care.

Domain	Outcome	Outcome Description
Infant	Growth	The infant shows appropriate progress in physical growth (weight, length, and head circumference) for their age.
Infant	Infant pain and stress	The presence of acute or chronic pain and stress in the infant, assessed through behavioral indicators (crying, facial expressions) or physiological indicators (changes in vital signs, e.g., heart rate or respiratory rate changes), often due to medical procedures or illness.
Infant	Nosocomial infection	An infection acquired during hospitalization, often due to prolonged NICU stay or use of invasive devices.
Infant	Length of NICU stay	The total number of days the infant spends specifically in the NICU before being discharged or transferred.
Parent	Bonding with infant	Development of emotional attachment to the baby.
Parent	Participation in care	Parents’ involvement in providing care for their infant.
Parent	Parental readiness for discharge	Parents’ preparedness to care for their baby at home after NICU discharge.
Parent	Stress	Emotional strain experienced by parents.
Parent	Shared decision-making	Parents and staff making care decisions together.
Parent	Parental knowledge of infants’ care and treatment	Parents’ understanding of their baby’s care, treatment, and medical needs.

## Data Availability

The datasets generated and analyzed during the study are available from the corresponding author on reasonable request due to ethical and privacy restrictions.

## Supplementary Materials

The following supporting information can be downloaded at: https://www.mdpi.com/article/10.3390/children13070862/s1, Figure S1: Descriptive importance rating scale provided to participants during the eDelphi survey; Figure S2: Example of feedback provided to participants in rounds 2 and 3; Table S1: Parent-related outcomes that reached consensus (≥70% rated as critically important [7,8,9]) in round 3 of the eDelphi survey, ranked from highest to lowest total percentage. Table S2: Infant-related outcomes that reached consensus (≥70% rated as critically important [7,8,9]) in Round 3 of the eDelphi survey, ranked from highest to lowest total percentage; Table S3: Healthcare professional-related outcomes that reached consensus (≥70% rated as critically important [7,8,9]) in round 3 of the eDelphi survey, ranked from highest to lowest total percentage; Table S4: Distributions of Scores Across Delphi Rounds (Total and by Group); Table S5: Voting Results and decisions from the consensus meeting.

## Abbreviations

The following abbreviations are used in this manuscript:

FCC	Family-Centered Care
NICU	Neonatal Intensive Care Units
COS	Core Outcome Set
COUSIN	Core Outcome Set and Outcome Measures of Family-Centered Care in Neonatal Care
GFCNI	Global Foundation for the Care of Newborn Infants
COMET	Core Outcome Measures Effectiveness Trials
COS-STAD	Core Outcome Set—Standards for Development
COS-STAR	Core Outcome Set—Standards for Reporting
GRADE	Grading of Recommendations Assessment, Development, and Evaluation
COINN	Council of International Neonatal Nurses
ESPNIC	European Society of Pediatric and Neonatal Intensive Care
ESPR	European Society of Pediatric Research
JISC	Joint Information Systems Committee
