# Supplementary material for: A Core Outcome Set for Family-Centered Care in Neonatal Intensive Care Settings: An International eDelphi Study and Online Consensus Meeting
Source: Children (Basel). 2026 Jun 29;13(7):862. doi: 10.3390/children13070862 (PMC13407020; doi:10.3390/children13070862)
Supplement: Supplementary file 1 [file children-13-00862-s001.zip › children-4336567-supplementary.pdf]

Electronic Supplemental Materials

Supplemental Material Figure S1. Descriptive importance rating scale provided to participants in the eDelphi survey

**Rating**	**Description**
1	Not at all important
2	Slightly important
3	Somewhat important
4	Moderately important
5	Important
6	Very important
7	Extremely important
8	Critically important
9	Absolutely essential
Unable to score	I do not feel able to assess this outcome

Supplemental Material Figure S2. Example of feedback provided to participants in rounds 2 and 3.

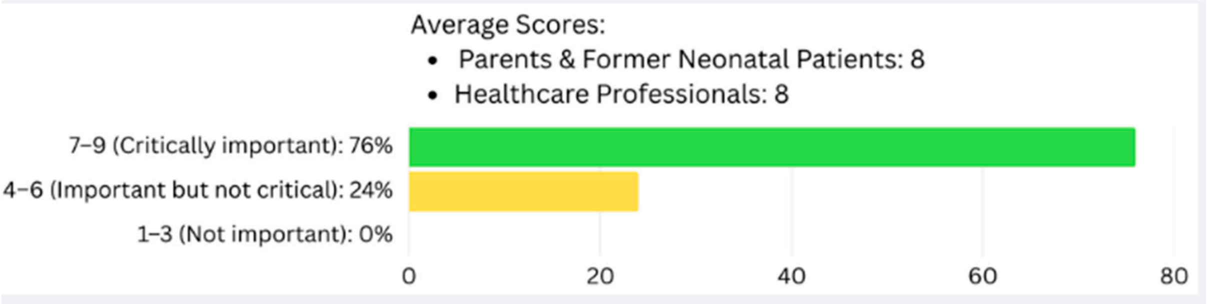

Supplemental Material Table S1. Parent-related outcomes that reached consensus (≥70% rated as critically important [7–9]) in round 3 of the eDelphi survey, ranked from highest to lowest total percentage

Outcome Name	Outcome Description	Total %	Parents & Former Neonatal Patients %	Healthcare Professionals %
Bonding with infant	Development of emotional attachment to the baby	97%	97%	98%
Parent-infant interaction	Meaningful contact and communication between parent and baby	95%	97%	93%
Participation in care	Parents' involvement in providing care for their infant	93%	93%	93%
Duration of skin-to skin contact	Length of time holding the baby against the parent's bare chest	93%	100%	89%
Parental competence of caring	Parents' ability to care for their baby	92%	100%	87%
Parental presence	Time physically spent with the baby in the NICU	91%	93%	89%
Parental trust in staff	Parents' confidence in the NICU staff's ability to care for their baby	91%	97%	87%
Parental confidence in caring	Parents' belief in their ability to provide care for their baby	89%	90%	89%
Parental readiness for discharge	Parents' preparedness to care for their baby at home after NICU discharge	89%	87%	91%
Physically holding the infant	Parents holding their baby in their arms	89%	100%	82%
Stress	Emotional strain experienced by parents	88%	97%	82%
Parent-staff communication	Exchange of information between parents and NICU staff	85%	90%	82%
Shared decision making	Parents and staff making care decisions together	84%	97%	76%
Parental knowledge of infants' care and treatment	Parents' understanding of their baby's care, treatment, and medical needs	84%	97%	76%
Self-efficacy	Parents' confidence in their ability to care for their baby	83%	83%	82%
Anxiety	Persistent worry or nervousness related to the baby's condition	81%	80%	82%
Parental autonomy	Parents' ability to make decisions about their baby's care	81%	87%	78%
Separation	Being apart from the infant during care in the NICU	80%	83%	78%
Parent understanding of infant development	Parents' knowledge of their baby's growth, abilities, and developmental needs	77%	83%	73%
Depression	Symptoms of low mood or clinical depression in parents	77%	77%	78%
Parental perception of staff support	Parents' views on the emotional, informational, and practical support provided by NICU staff	75%	87%	67%
Perceived parental role (maternal or paternal)	Parents' understanding and perception of their role as a mother or father	73%	77%	71%
Coping with emotions	Parents' ability to manage and adapt to emotional challenges	73%	83%	67%
Parental satisfaction with care	Parents' contentment with the care provided to their baby and themselves during NICU admission	72%	83%	64%
Post-traumatic stress disorder	Ongoing psychological distress or trauma-related symptoms in parents	70%	77%	66%

Supplemental Material Table S2. Infant-related outcomes that reached consensus (≥70% rated as critically important [7–9]) in round 3 of the eDelphi survey, ranked from highest to lowest total percentage

Outcome Name	Outcome Description	Total %	Parents & Former Neonatal Patients %	Healthcare Professionals %
Infant pain	The presence of acute or chronic pain in the infant, assessed through behavioral (crying, facial expressions) or physiological (changes in vital signs) indicators, often due to medical procedures or illness.	95%	93%	96%
Neurodevelopment	The progression of brain and nervous system functions in the infant, reflected in the acquisition of cognitive, motor, language, social-emotional, and adaptive skills	93%	93%	93%
Infant stress	Physiological (heart rate changes, respiratory rate changes) or behavioral (grimacing, crying) signs indicating that the infant is experiencing discomfort, pain, or distress.	92%	93%	91%
Comfort of infant	A state in which the infant shows minimal or no signs of discomfort, pain, or distress, as indicated by calm behavior, stable physiological parameters, and relaxed body posture	85%	77%	91%
Infant sleep quality	The extent to which the infant experiences restful, undisturbed, and developmentally appropriate sleep patterns	81%	70%	84%
Intraventricular hemorrhage	Bleeding into the brain's ventricular system, common in preterm infants, may lead to brain injury and long-term developmental challenges depending on severity	75%	75%	76%
Growth	The infant shows appropriate progress in both physical growth (weight, length, and head circumference) for appropriate age.	73%	73%	73%
Nosocomial infection	An infection acquired during hospitalization, often due to prolonged NICU stay or use of invasive devices	73%	71%	73%
Sepsis	A systemic infection in the infant, often bacterial, that triggers a widespread inflammatory response	72%	77%	69%
Length of NICU stay	The total number of days the infant spends specifically in the NICU before being discharged or transferred	71%	73%	69%
Mortality	The death of the infant during the neonatal period or prior to discharge	71%	83%	62%

Supplemental Material Table S3. Healthcare professional-related outcomes that reached consensus (≥70% rated as critically important [7–9]) in round 3 of the eDelphi survey, ranked from highest to lowest total percentage

Outcome Name	Outcome Description	Total %	Parents & Former Neonatal Patients %	Healthcare Professionals %
Staff competency	The knowledge, skills, and professional abilities of healthcare professionals to provide safe, effective, and evidence-based care to infants and families in the NICU.	97%	93%	100%
Staff training and knowledge of family-centered care	The education, skills, and understanding that healthcare professionals possess regarding FCC principles and practices, enabling them to effectively involve and support families in the care of their infants in the NICU.	93%	93%	93%
Staff relationship with parents	The quality and nature of interactions between healthcare professionals and parents, including communication, trust, collaboration, and mutual respect in the infant's care.	91%	90%	91%
Staff attitudes	The beliefs, perceptions, and approaches of healthcare professionals toward infants, parents, and care practices, as reflected in their communication, behavior, and interactions in neonatal intensive care units.	89%	93%	87%
Staff confidence	The self-assurance and perceived competence of healthcare professionals in their knowledge, skills, and decision-making when providing care to infants and families in the NICU.	89%	83%	93%
Staff workload	The amount and intensity of tasks, responsibilities, and patient care activities assigned to healthcare professionals in the NICU	89%	87%	91%
Staff responsiveness to parental needs	The promptness, attentiveness, and appropriateness with which healthcare professionals recognize and address parents' concerns, questions, and emotional or practical needs in the NICU.	87%	93%	82%
Staff health and well-being	The physical, mental, and emotional state of healthcare professionals working in the NICU.	76%	83%	71%
Delivery of continuity care	The consistent and coordinated provision of care to an infant and family by the same healthcare professionals or team across time and settings.	75%	77%	73%
Staff satisfaction	The degree to which healthcare professionals feel fulfilled, valued, and content with their work, roles, and work environment in the NICU.	73%	80%	69%
Staff turnover, intention to leave	The rate at which healthcare professionals leave their roles or express a desire to leave their current neonatal healthcare position.	72%	87%	62%

Supplemental Material Table S4. Distributions of Scores Across eDelphi rounds (Total and by Group)

		Delphi round 1 Result										Delphi round 2 Result											Delphi round 3 Result									
		Total Percentage (%)			HCP (%)			Parents & FNP (%)				Total Percentage (%)			HCPs (%)			Parents & FNP (%)				Total Percentage (%)			HCP (%)			Parents & FNP (%)			
Outcome Domain	Outcomes	1-3	4-6	7-9	1-3	4-6	7-9	1-3	4-6	7-9	Delphi r1 Consensus	1-3	4-6	7-9	1-3	4-6	7-9	1-3	4-6	7-9	Delphi r2 Consensus	1-3	4-6	7-9	1-3	4-6	7-9	1-3	4-6	7-9	Delphi R3 Consensus
**Parent**	Stress	0	25	75	0	32	68	0	14	86	IN	1	7	92	2	7	91	0	6	94	IN	1	11	88	2	16	82	0	3	97	IN
	Depression	0	29	71	0	33	67	0	21	79	IN	0	14	86	0	19	82	0	6	94	IN	0	23	77	0	22	78	0	23	77	IN
	Anxiety	0	25	75	0	26	75	0	26	74	IN	2	11	87	0	13	87	6	6	88	IN	1	17	81	2	16	82	0	20	80	IN
	Sleep deprivation	2	34	64	3	37	60	0	29	71	No Consensus	6	30	64	6	35	59	6	22	72	No Consensus	4	44	52	7	44	49	0	40	60	No Consensus
	Post-traumatic stress disorder	4	21	76	5	21	75	2	19	79	IN	1	24	74	2	30	69	0	16	84	IN	0	30	70	0	34	66	0	23	77	IN
	Anticipatory grief	5	35	60	7	34	60	3	34	63	No Consensus	5	39	56	4	47	49	7	26	68	No Consensus	3	47	50	4	58	38	0	28	72	No Consensus
	Self-blame	5	34	61	8	37	56	0	31	69	No Consensus	4	41	56	7	46	48	0	31	69	No Consensus	3	45	52	4	47	49	0	40	60	No Consensus
	Parental guilt	4	30	66	7	29	65	0	32	68	No Consensus	2	35	63	4	44	52	0	19	81	No Consensus	3	44	53	4	51	44	0	30	70	No Consensus
	Coping with emotions	1	24	76	2	25	73	0	19	81	IN	1	31	67	2	32	67	0	31	69	No Consensus	0	27	73	0	33	67	0	17	83	IN
	Coping through religious or spiritual practice	15	32	53	13	48	39	15	62	23	No Consensus	19	53	28	15	56	30	26	48	26	OUT										OUT
	Parental competence of caring	2	23	75	2	16	83	0	34	66	IN	0	12	88	0	11	89	0	13	88	IN	0	8	92	0	13	87	0	0	100	IN
	Parental autonomy	0	32	68	0	31	69	0	33	67	No Consensus	1	21	78	2	20	78	0	22	78	IN	0	19	81	0	22	78	0	13	87	IN
	Self-efficacy	3	34	64	3	30	67	0	40	60	No Consensus	1	27	72	2	28	70	0	26	74	IN	0	17	83	0	18	82	0	17	83	IN
	Parental confidence in caring	0	23	77	0	18	83	0	29	71	IN	0	15	85	0	15	85	0	16	84	IN	0	11	89	0	11	89	0	10	90	IN
	Perceived parental role (maternal or paternal)	3	24	73	2	23	76	5	24	71	IN	1	20	79	0	22	78	3	16	81	IN	0	27	73	0	29	71	0	23	77	IN
	Participation in care	0	13	87	0	13	87	0	12	88	IN	0	5	95	0	6	94	0	3	97	IN	0	7	93	0	7	93	0	7	93	IN
	Parent-infant interaction	0	5	95	0	2	98	0	7	93	IN	0	4	97	0	4	96	0	3	97	IN	0	5	95	0	7	93	0	3	97	IN
	Parental presence	3	5	93	2	5	94	2	5	93	IN	0	12	88	0	13	87	0	9	91	IN	0	9	91	0	11	89	0	7	93	IN
	Skin-to-skin contact	0	8	92	0	6	94	0	7	93	IN	1	8	91	2	9	89	0	6	94	IN	1	5	93	2	9	89	0	0	100	IN
	Physically holding the infant	0	18	83	0	19	81	0	15	85	IN	0	16	84	0	19	82	0	13	88	IN	3	9	88	4	13	82	0	0	100	IN
	Bonding with infant	0	6	94	0	5	95	0	7	93	IN	0	4	97	0	4	96	0	3	97	IN	0	3	97	0	2	98	0	3	97	IN
	Separation	5	22	73	3	22	74	8	23	70	IN	1	17	82	0	19	81	3	13	84	IN	1	19	80	2	20	78	0	17	83	IN
	Parental satisfaction with care	1	30	69	2	33	65	0	26	74	No Consensus	2	24	73	2	26	72	3	22	75	IN	0	29	71	0	36	64	0	17	83	IN
	Parent-staff communication	1	12	87	2	13	86	0	10	91	IN	0	8	92	0	7	93	0	9	91	IN	0	15	85	0	18	82	0	10	90	IN
	Shared decision-making	1	21	78	2	25	73	0	12	88	IN	0	14	86	0	17	83	0	9	91	IN	0	16	84	0	24	76	0	3	97	IN
	Parental perception of staff support	3	29	68	2	27	71	2	33	64	No Consensus	0	26	74	0	28	72	0	22	78	IN	0	25	75	0	33	67	0	13	87	IN
	Parental trust in staff	1	14	85	2	14	84	0	12	88	IN	0	11	90	0	11	89	0	9	91	IN	0	9	91	0	13	87	0	3	97	IN
	Parental knowledge of infants' care and treatment	1	26	74	2	29	70	0	19	81	IN	0	16	84	0	22	78	0	6	94	IN	0	16	84	0	24	76	0	3	97	IN
	Parental preparation for NICU admission	5	46	49	6	54	40	2	36	62	No Consensus	4	50	47	6	54	41	0	44	56	No Consensus	6	51	44	9	56	36	0	43	57	No Consensus
	Parental readiness for discharge	0	17	83	0	13	87	0	24	76	IN	1	7	92	2	4	94	0	13	88	IN	0	11	89	0	9	91	0	13	87	IN
	Parental ability to advocate for their baby	1	21	78	2	22	76	0	19	81	IN	0	23	77	0	24	76	0	22	78	IN	0	13	87	0	13	87	0	13	87	IN
	Parental comfort	1	39	60	0	39	61	2	38	60	No Consensus	4	44	52	4	44	52	3	44	53	No Consensus	1	45	53	2	51	47	0	33	67	No Consensus
	Quality of life	4	25	71	7	30	64	0	20	81	IN	0	22	78	0	26	74	0	16	84	IN	1	33	65	2	38	60	0	27	73	No Consensus
	Confidence in breastfeeding	4	30	67	5	29	67	2	29	68	No Consensus	2	31	66	2	30	69	3	34	63	No Consensus	1	37	61	0	44	56	3	27	70	No Consensus
	Perceived support from family and friends	7	50	43	8	49	42	5	52	43	No Consensus	4	48	49	6	50	44	0	44	56	No Consensus	4	64	32	7	62	31	0	63	37	No Consensus
	Perceived family functioning	3	42	55	5	47	48	0	34	66	No Consensus	2	51	47	4	49	47	0	53	47	OUT										
	Use of milk bank											17	49	34	24	46	30	3	53	43	OUT										
	Peer support											5	47	49	6	57	37	3	28	69	No Consensus	1	44	55	2	56	42	0	23	77	No Consensus
	Parent understanding of infant development											0	27	73	0	28	72	0	25	75	IN	0	23	77	0	27	73	0	17	83	IN
**Infant**	Mortality	14	18	67	21	21	58	2	15	83	No Consensus	6	29	65	7	35	57	3	19	78	No Consensus	5	24	71	9	29	62	0	17	83	IN
	Necrotizing enterocolitis	14	31	55	18	31	51	5	32	62	No Consensus	8	31	61	11	39	50	3	17	80	No Consensus	5	45	50	9	42	49	0	45	55	No Consensus
	Weight gain	2	34	64	0	36	65	2	32	66	No Consensus	2	28	70	4	28	69	0	28	72	IN	1	41	57	2	42	56	0	40	60	No Consensus
	Breastfeeding	4	25	71	0	23	77	7	29	64	IN	4	31	65	4	32	65	3	31	66	No Consensus	3	40	57	2	44	53	3	33	63	No Consensus
	Formula feeding	15	51	34	20	46	34	8	58	35	No Consensus	13	65	22	17	65	17	7	63	30	OUT										
	Combination feeding methods (Breast, formula and bottle)	12	41	47	15	47	39	5	34	61	No Consensus	6	57	37	8	60	32	3	50	47	OUT										
	Gastric tube feeding	13	45	42	16	48	36	5	41	54	No Consensus	6	52	42	7	61	32	3	37	60	No Consensus	6	58	37	9	60	31	0	54	46	No Consensus
	Time to full gastrointestinal feeding	7	46	48	8	44	48	3	49	49	No Consensus	6	52	42	8	49	43	3	57	40	OUT										
	Duration of nasogastric tube retention	13	53	34	15	53	33	8	54	38	No Consensus	10	58	32	13	53	34	3	67	30	OUT										
	Duration of total enteral nutrition	15	44	41	18	43	38	8	46	46	No Consensus	10	54	36	11	53	36	7	57	37	OUT										
	Feeding intolerance	15	42	43	17	47	37	10	36	54	No Consensus	9	47	44	13	52	35	3	39	58	No Consensus	7	51	43	11	47	42	0	27	73	No Consensus
	Growth and development	3	13	84	2	15	84	2	12	86	IN	0	11	90	0	13	87	0	7	94	IN	3	24	73	4	22	73	0	39	61	IN
	Duration of oxygen therapy	12	24	65	13	28	58	7	17	76	No Consensus	5	25	71	8	28	64	0	19	81	IN	3	40	58	4	40	56	0	43	57	No Consensus
	Bronchopulmonary dysplasia	15	28	57	20	31	49	3	25	72	No Consensus	7	31	61	11	32	57	0	30	70	No Consensus	4	44	51	7	46	48	0	38	62	No Consensus
	Retinopathy of prematurity	20	29	51	25	33	42	10	23	67	No Consensus	6	34	60	9	34	57	0	34	66	No Consensus	4	44	51	7	47	47	0	29	71	No Consensus
	Resuscitation	17	25	58	20	32	48	8	14	78	No Consensus	8	29	63	13	28	56	0	29	71	No Consensus	7	29	64	11	27	62	0	7	93	No Consensus
	Neurodevelopment	2	10	86	2	7	92	0	15	85	IN	0	6	94	0	6	94	0	6	94	IN	0	7	93	0	7	93	0	23	77	IN
	Sepsis	6	27	68	7	33	61	3	18	80	No Consensus	2	24	74	4	23	74	0	25	75	IN	4	24	72	7	24	69	0	25	75	IN
	Intraventricular hemorrhage	13	27	60	14	34	53	8	17	75	No Consensus	2	26	71	4	28	68	0	23	77	IN	3	22	75	4	20	76	0	29	71	IN
	Nosocomial infection	7	30	63	7	34	53	3	23	73	No Consensus	2	35	63	2	41	57	3	24	72	No Consensus	3	25	73	4	22	73	0	33	67	IN
	Length of hospital stay	4	26	71	3	24	73	3	29	69	IN	1	21	78	2	22	76	0	19	81	IN	1	31	68	2	29	69	0	27	73	No Consensus
	Length of NICU stay	4	25	71	4	24	73	2	26	71	IN	1	22	77	2	26	72	0	16	84	IN	3	27	71	4	27	69	0	37	63	IN
	Readmission	2	39	59	2	38	60	3	40	58	No Consensus	2	27	71	2	30	69	3	22	75	IN	1	32	67	2	29	69	0	27	73	No Consensus
	Hospital expenses	25	38	38	34	31	34	8	49	44	No Consensus	18	48	34	22	52	26	10	42	48	OUT										
	Duration of antibiotic use	21	35	43	32	27	41	5	46	49	No Consensus	13	54	33	19	45	36	3	68	29	OUT										

	Infiltration of peripheral intravenous	20	43	37	25	44	32	10	42	48	No Consensus	17	54	29	25	46	29	3	67	30	OUT										
	Severity of illnesses	11	29	60	16	31	53	5	27	68	No Consensus	6	27	67	9	28	63	0	26	74	No Consensus	1	31	68	2	33	64	0	7	63	No Consensus
	Infant stress	11	40	50	17	44	39	0	32	68	No Consensus	1	16	83	2	17	82	0	16	84	IN	0	8	92	0	9	91	0	7	93	IN
	Infant pain											0	16	84	0	17	83	0	16	84	IN	0	5	95	0	4	96	0	43	57	IN
	Exclusive Breastfeeding at discharge											9	49	42	7	54	39	13	41	47	OUT										
	Use of non-pharmacological measures (massage, music)											12	43	45	13	46	41	9	38	53	No Consensus	4	36	60	7	29	64	0	43	57	No Consensus
	Comfort of infant											1	15	84	2	13	85	0	19	81	IN	0	15	85	0	9	91	0	23	77	IN
	Infant sleep quality											1	29	69	2	28	70	0	32	68	No Consensus	1	17	81	2	13	85	0	23	77	IN
	Hypoxic ischemic encephalopathy											14	30	57	15	34	51	11	21	68	No Consensus	4	41	55	7	44	49	0	32	68	No Consensus
**Staff**	Staff attitudes	2	21	77	0	21	79	2	21	76	IN	0	11	90	0	11	89	0	9	91	IN	0	11	89	0	13	87	0	7	93	IN
	Staff confidence	1	20	79	0	22	78	0	17	83	IN	0	11	90	0	11	89	0	9	91	IN	0	12	88	0	7	93	0	17	83	IN
	Staff relationship with parents	1	14	85	0	15	86	0	14	86	IN	0	13	87	0	15	85	0	9	91	IN	0	9	91	0	9	91	0	10	90	IN
	Staff workload	1	18	81	2	16	83	0	19	81	IN	1	9	90	0	9	91	3	9	88	IN	0	11	89	0	9	91	0	13	87	IN
	Staff competency	0	15	85	0	19	81	0	7	93	IN	0	9	91	0	9	91	0	9	91	IN	0	3	97	0	0	100	0	7	93	IN
	Staff responsiveness to parental needs	0	22	78	0	21	79	0	21	79	IN	1	19	80	0	22	78	3	13	84	IN	0	13	87	0	18	82	0	7	93	IN
	Staff satisfaction	5	30	66	5	32	64	2	26	71	No Consensus	1	27	72	2	28	70	0	25	75	IN	1	25	73	2	29	69	0	20	80	IN
	Delivery of continuity care	2	20	78	2	22	76	0	18	82	IN	0	16	84	0	17	83	0	16	84	IN	0	25	75	0	27	73	0	23	77	IN
	Staff health and well-being											2	29	69	4	30	67	0	28	72	No Consensus	1	23	76	2	27	71	0	17	83	IN
	Staff training and knowledge of family-centered care											1	9	90	2	13	85	0	3	97	IN	0	7	93	0	7	93	0	7	93	IN
	Staff turnover, intention to leave											5	27	69	7	28	65	0	25	75	No Consensus	4	24	72	4	33	62	3	10	87	IN

Electronic Supplemental Material Table S5. Voting Results and decisions from the consensus meeting

Outcome Domain	Outcome Name	round 1 yes %	r1 No	r1 Yes	Discussion	round 2 yes %	r2 No	r2 Yes	Discussion	Final Decision
Parent	Bonding with infant	90	1	9	Included	100	0	10		Included
Staff	Staff competency	50	5	5	Excluded					
Parent	Parent-infant interaction	60	4	6	Excluded					
Infant	Infant pain	80	2	8	Combined with infant stress following discussion	88	1	7		Included; combined with infant stress
Staff	Staff training and knowledge of family-centered care	80	2	8	Included	25	6	2	No further action following discussion	Excluded; threshold not reached
Parent	Participation in care	80	2	8	Included	100	0	8		Included
Infant	Neurodevelopment	40	6	4	Excluded					
Parent	Duration of skin-to skin contact	70	3	7	Excluded					
Parent	Parental competence of caring	50	5	5	Excluded					
Infant	Infant stress	90	1	9	Combined with infant pain following discussion					
Parent	Parental presence	70	3	7	Retained for second round voting following discussion	78	2	7		Excluded; threshold not reached
Parent	Parental trust in staff	0	10	0	Excluded					
Staff	Staff relationship with parents	30	7	3	Excluded					
Parent	Parental confidence in caring	70	3	7	Excluded					
Parent	Parental readiness for discharge	80	2	8	Included	67/90	3/1	6/9	Initial revote did not reach the predefined threshold (67% agreement). Following further discussion, participants requested an additional revote)	Included following additional revote (90%)
Staff	Staff attitudes	20	8	2	Excluded					
Staff	Staff confidence	10	9	1	Excluded					
Staff	Staff workload	20	8	2	Excluded					
Parent	Physically holding the infant	30	7	3	Excluded					
Parent	Stress	100	0	10	Included	100	0	10		Included
Staff	Staff responsiveness parental needs	20	8	2	Excluded					
Infant	Comfort of infant	60	4	6	Excluded					
Parent	Parent-staff communication	80	2	8	Included	50	5	5	No further action following discussion	Excluded
Parent	Shared decision making	90	1	9	Included	100	0	10		Included
Parent	Parental knowledge of infants' care and treatment	80	2	8	Included	90	1	9		Included
Parent	Self-efficacy	50	5	5	Excluded					
Parent	Anxiety	50	5	5	Excluded					
Parent	Parental autonomy	10	9	1	Excluded					
Infant	Infant sleep quality	40	6	4	Excluded					
Parent	Separation	20	8	2	Excluded					
Parent	Parent understanding of infant development	60	4	6	Excluded					
Parent	Depression	40	6	4	Excluded					
Staff	Staff health and well-being	20	8	2	Excluded					
Staff	Delivery of continuity care	0	10	0	Excluded					
Parent	Parental perception of staff support	40	6	4	Excluded					
Parent	Perceived parental role (maternal or paternal)	20	8	2	Excluded					
Parent	Coping with emotions	20	8	2	Excluded					
Infant	Intraventricular hemorrhage	10	9	1	Excluded					
Infant	Growth	80 (70)	2(3)	8(7)	One participant amended her/his vote from no to yes	80	2	8		Included
Staff	Staff satisfaction	0	10	0	Excluded					
Infant	Nosocomial infection	80	2	8	Included	90	1	9		Included
Infant	Sepsis	40	6	4	Excluded					
Staff	Staff turnover, intention to leave	0	10	0	Excluded					
Parent	Parental satisfaction with care	40	6	4	Excluded					
Infant	Length of NICU stay	100	0	10	Included	100	0	10		Included
Infant	Mortality	50	5	5	Excluded					
Parent	Post-traumatic stress disorder	70	3	7	Excluded					

Abbreviations: N = number of No votes; Y = number of Yes votes.
